# Biochemical Pathways Delivering Distinct Glycosphingolipid Patterns in MDA-MB-231 and MCF-7 Breast Cancer Cells

**DOI:** 10.3390/cimb46090608

**Published:** 2024-09-15

**Authors:** Anita Markotić, Jasminka Omerović, Sandra Marijan, Nikolina Režić-Mužinić, Vedrana Čikeš Čulić

**Affiliations:** 1 Department of Medical Chemistry and Biochemistry, University of Split School of Medicine, 21000 Split, Croatia; anita.markotic@mefst.hr (A.M.); sandra.marijan@mefst.hr (S.M.); nikolina.rezic.muzinic@mefst.hr (N.R.-M.); 2 Department of Immunology, University of Split School of Medicine, 21000 Split, Croatia; jasminka.omerovic@mefst.hr

**Keywords:** glycosphingolipids, growth factor receptors, breast cancer, structure, membrane lipid remodeling

## Abstract

The complex structure of glycosphingolipids (GSLs) supports their important role in cell function as modulators of growth factor receptors and glutamine transporters in plasma membranes. The aberrant composition of clustered GSLs within signaling platforms, so-called lipid rafts, inevitably leads to tumorigenesis due to disturbed growth factor signal transduction and excessive uptake of glutamine and other molecules needed for increased energy and structural molecule cell supply. GSLs are also involved in plasma membrane processes such as cell adhesion, and their transition converts cells from epithelial to mesenchymal with features required for cell migration and metastasis. Glutamine activates the mechanistic target of rapamycin complex 1 (mTORC1), resulting in nucleotide synthesis and proliferation. In addition, glutamine contributes to the cancer stem cell GD2 ganglioside-positive phenotype in the triple-negative breast cancer cell line MDA-MB-231. Thieno[2,3-*b*]pyridine derivative possesses higher cytotoxicity against MDA-MB-231 than against MCF-7 cells and induces a shift to aerobic metabolism and a decrease in S(6)nLc4Cer GSL-positive cancer stem cells in the MDA-MB-231 cell line. In this review, we discuss findings in MDA-MB-231, MCF-7, and other breast cancer cell lines concerning their differences in growth factor receptors and recent knowledge of the main biochemical pathways delivering distinct glycosphingolipid patterns during tumorigenesis and therapy.

## 1. Introduction

The elucidation of the mechanisms involved in metabolic rewiring is an important milestone for understanding tumorigenesis with the aim of developing effective cancer treatment [1]. Recently, we described a novel thieno[2,3-*b*]pyridine anticancer compound that lowers the triple-negative cancer stem cell (CSC) fraction in the MDA-MB-231 cell line. A mix of both CSC and non-CSC subpopulations shows a metabolic shift [2]. Treatment with thieno[2,3-*b*]pyridine derivative changes the distinct glycosphingolipid (GSL) pattern in MDA-MB-231 and MCF-7 breast cancer cell lines. These lines differ concerning the expression of human epidermal growth factor receptor 3 (HER3), being both present in [3,4] and absent in different cells. The metabolic profiling of two different breast cancer cell lines reveals the inverse effect of thieno[2,3-*b*]pyridine derivative on two metabolites: lactate and glycerylmonostearate decrease in MDA-MB-231 and increase in MCF-7 cells [2]. Therefore, in this review, we aim to explore and discuss the recent knowledge of GSL expression in breast cancer cells and the methods of cancer treatment by targeting its GSLs. We specifically point out the treatment of breast cancer cells with thieno[2,3-*b*]pyridine compounds. They were discovered via virtual screening targeting phosphoinositide-specific phospholipase C (pi-PLC) [5]. Their antitumor activity was proven in numerous tumor cell lines, where they inhibited the growth of triple-negative breast cancer cell lines at nanomolar concentrations [6,7].

## 2. The Role of Glycosphingolipids in Membrane Lipid Rafts Is Derived from Their Structure

**Figure 1:**
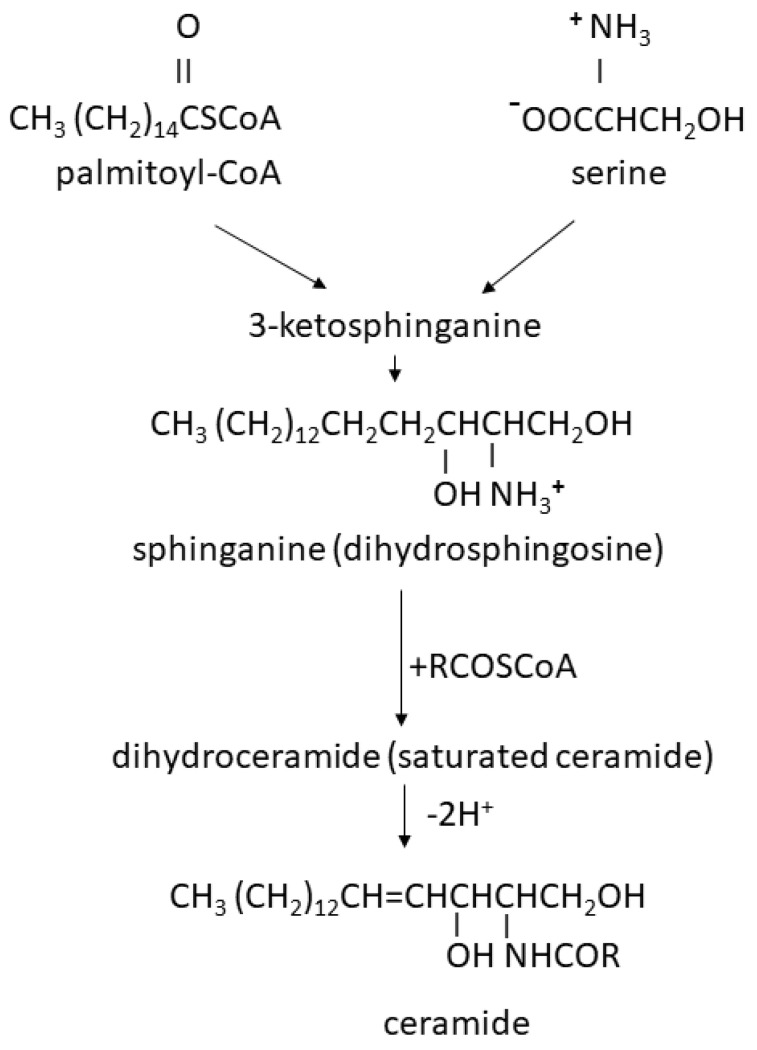
Ceramide synthesis.

Glycosphingolipids are essential constituents of the plasma membrane outer leaflet, exposing their sugar residues as their hydrophilic portion in the extracellular space. Their synthesis begins with dihydrosphingosine synthesized from activated palmitic acid (palmitoyl-CoA) [8] and, rarely, stearic acid [9], which binds the amino acid serine, which has a branch chain with an -OH terminal group (Figure 1). Therefore, dihydrosphingosine is an amino alcohol. It binds the -CO group in acyl-CoA (palmitoyl or stearoyl, RCOSCoA) with an amide bond, forming saturated ceramide (dihydroceramide). Dihydroceramide is dehydrogenated within its dihydrosphingosine part, resulting in ceramide containing the long-chain unsaturated amino alcohol sphingosine (Figure 1).

The cellular level of ceramide is modulated through the ubiquitination of N-acylsphingosine amidohydrolase 1, which cleaves the acyl residue (represented as COR within the -NHCOR ceramide group in Figure 1) from ceramide. Thereby, ubiquitin domain-containing protein 1 (UBTD1), which performs ubiquitination, indirectly limits the self-phosphorylation of epidermal growth factor receptor (EGFR) by modulating the ceramide balance through the degradation of the N-acylsphingosine amidohydrolase 1 [10]. Specifically, the ceramide level directly affects the GM3 level of GSL, which is a well-known inhibitor of EGFR signal transduction [10].

**Figure 2:**
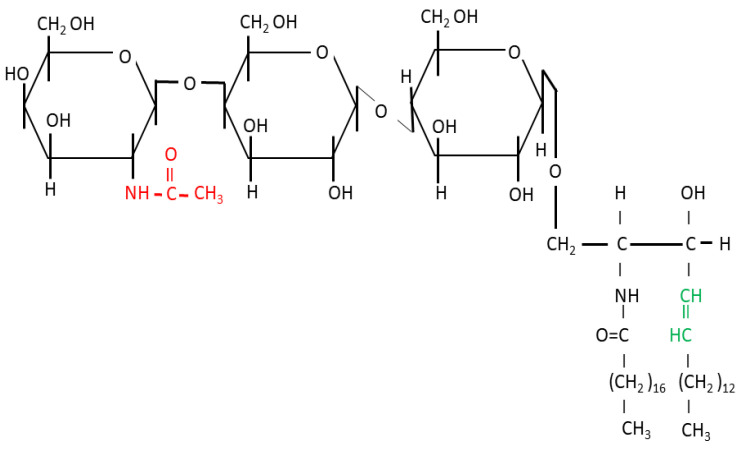
Structure of glycosphingolipid Gg3Cer. Acetamide group of N-acetylglucosamine is marked in red. *Trans* double bond within sphingosine, responsible for lipid raft formation, is marked in green.

The binding of sugar residues to ceramide by a glycosidic bond forms glycosphingolipids [11]. Many functions of GSLs are due to their capability to form hydrogen-bound clusters using hydroxyl and acetamide groups from their sugar residues [12,13]. A neutral GSL Gg3Cer, consisting of lactose and N-acetylglucosamine, is shown in Figure 2. It is called neutral due to its inability to release or accept hydrogen ions in a physiological pH medium.

On the other hand, acidic GSLs, named gangliosides, contain N-acetylneuraminic acid (Neu5Ac, also called sialic acid) as the third sugar instead of N-acetylglucosamine in Gg3Cer (Figure 2) and release hydrogen ions, leaving negatively charged N-acetyneuraminate residues. The N-acetyneuraminate-lactose part of ganglioside GM3 is essential for the binding of gangliosides to the extracellular domain of EGFR, modulating its activity [14]. The interaction of GSL with other sphingolipids, cholesterol, and transmembrane proteins, including receptors or signal transducers, forms lipid rafts [15]. The hydrophobic ceramide part anchors GSL within the membrane. In sphingolipids, saturated acyl chains such as palmitic and stearic acid are the main components and are present (at least in the nervous system) in sphingolipid-enriched domains and lipid rafts, packed tightly in the hydrophobic core of a bilayer mainly with saturated glycerophospholipid dipalmitoylphosphatidylcholine [16]. In humans with healthy diets, glycerophospholipids with unsaturated fatty acids, which are outside of lipid rafts, contain exclusively *cis*-fatty acids. Hydrogen ions are on the same side of their double bonds, which causes the carbon chain to bend, thereby causing higher membrane fluidity [17]. The sphingosine part of the sphingolipid contains a *trans* double bond (Figure 2). Hydrogens are on the opposite sides of the double bond, causing the carbon chain to remain straight and enabling tight packaging within lipid rafts. The introduction of *cis* conformation into the fatty acyl tail of sphingolipids disrupts the lipid raft ordered domains, whereas *trans* fatty acid conformation promotes raft formation [18]. 

## 3. Different Plasma Membrane Lipids Influence Growth Receptor Function

Neoplastic growth of breast cancer cells often relies on the overactivity of members of the ErbB tyrosine kinase receptor family [19]. High expression and/or gene mutations in *ErbB1*/*EGFR*, *ErbB2*, *ErbB3*, and *ErbB4* constitutively activate receptor kinase signaling [20]. When such a biological event occurs, it generally fosters failure in cell physiology and eventually drives tumor phenotype development. Hence, these receptors have been considered targets of particular interest for therapeutic intervention. In healthy cells, the activity of these receptors is driven by their ligands. When bound to the receptor, each ligand triggers a seemingly overlapping signaling pathway [21]. On the cell surface, the process of signal transduction across the plasma membrane is linked to conformational rearrangement of each receptor domain (extracellular ligand binding, transmembrane, juxtamembrane, and tyrosine kinase) and higher-order assemblies (dimers of EGF-induced dimers) [22]. 

Over the past 30 years, the complex conformational landscape of these receptors has been described, and the structure–activation interdependency has become more clearly understood [23,24]. Because of these well-characterized ligand–receptor models [25] and their prominent role in cancer, considerable drug therapy tools have been conceptualized in an attempt to hamper receptor activity [26]. A range of antibodies, allosteric inhibitors, and cyclic peptides have been largely used for therapy, either by preventing a dynamic dimer state, stabilizing an inactive conformation, or targeting sites different from the orthosteric and highly conserved ATP-binding site [27,28,29,30,31]. 

The role of the plasma membrane lipid environment is of particular interest and is increasingly being explored, considering the clinical significance of heterogeneous and highly dynamic membrane lipid content in cancer cells [32]. Advances pursued in a number of comparative studies, including molecular dynamic simulation, structural modeling, and equally important biophysical and computational studies on lipid-dependent receptor signaling are unfolding and genuinely challenge well-established linear and simple models of receptor activation. It should be noted that the biological role of lipids in this process is not yet well understood and remains incomplete. To date, the emerging picture shows that highly dynamic membrane lipid environment content paradoxically shapes receptor signaling in healthy and cancer cells [33,34]. Because of lipid metabolism, the dynamic membrane environment changes and participates in ligand–receptor signaling. For example, the metabolic enzyme lysophosphatidylcholine acyltransferase, LPCAT1, which is involved in the biosynthetic pathway that controls membrane phospholipid saturation, prevents the internalization and degradation of the receptor and holds the receptors on the cell surface, thereby providing the input for tumor formation and cancer progression [35]. 

Membrane lipids are involved in the reorganization of signaling molecules in lipid microdomain rafts and structural fluctuations in the conformational landscape of membrane proteins [36,37,38]. As revealed by several structural studies of full-length receptors, surrounding lipids particularly change the dimerization propensity, stabilization, and orientation of juxtamembrane and kinase domains of receptors [39,40]. For example, phosphatidylinositol 4,5-bisphosphate (PIP_2_) regulates transition of the inactive to the active dimer conformers. Under steady-state conditions, PIP_2_ membrane acidic lipids, which are highly concentrated in the raft microdomains, interact with the juxtamembrane domain of the receptor and likely stabilize the inactive EGFR dimer. In response to ligand stimulation, positive cooperation between the domains occurs, and the receptor dimer immediately becomes active and displays short-lived kinase activity. Signaling molecules involved in receptor downstream signaling such as protein kinase C (PKC) and membrane-bound phospholipase C gamma (PLCγ) induce phosphorylation in Thr654 and hydrolysis of PIP_2_, respectively. These events lead to juxtamembrane helix dimer dissociation, and the whole structure of the receptor returns to the initial, inactive state [41,42]. 

In addition, ErbB receptors interact with other species of plasma lipids, such as glycosphingolipid GM3. Differences in the interaction energies and dynamics of PIP_2_ and GM3 with receptors revealed possible differential modes of receptor structure and function mediation [43]. The retention of ErbB2 within the plasma membrane is due to lipid raft microdomains. Protein MAL2 mediates lipid raft formation, leading to ErbB2 plasma membrane retention and enhanced ErbB2 signaling in breast cancer cells [44]. EGF-induced lipid raft localization of the EGFR-ErbB2 heterodimer is crucial for Akt phosphorylation and breast cancer cell proliferation [45].

Interaction with other membrane lipids, such as cholesterol, which constitutes half of the functional membrane component, has been observed. With advancements in NMR spectroscopy, a tool for lipid membrane investigation, and molecular dynamics studies, a detailed description of the exact mechanistic understanding of cholesterol contribution in the dynamic modulation and rearrangement of the receptor has been reported [46]. The cholesterol-recognition/amino acid consensus (CRAC) sequence within transmembrane and juxtamembrane segments contains two overlapping motifs (A 661-XXX-G 665 and I 659-XXX-V663). It was observed that at high concentrations of cholesterol (16% *w/w*), the interactions between these two motifs are disrupted, helix–helix dimers fall apart, and the whole transmembrane segments separate [47]. 

Finally, the propensity of different plasma membrane lipids to subtly modify dimer conformity and dynamics presents prospects for new, attractive cancer drug therapy. In general, the gradual profiling of the membrane environment will likely contribute to a better understanding of both physiological and pathological lipid-dependent remodeling signaling pathways. These results may point to the need to prioritize lipid cell-profiling before studying receptor signaling models in general.

**Figure 3:**
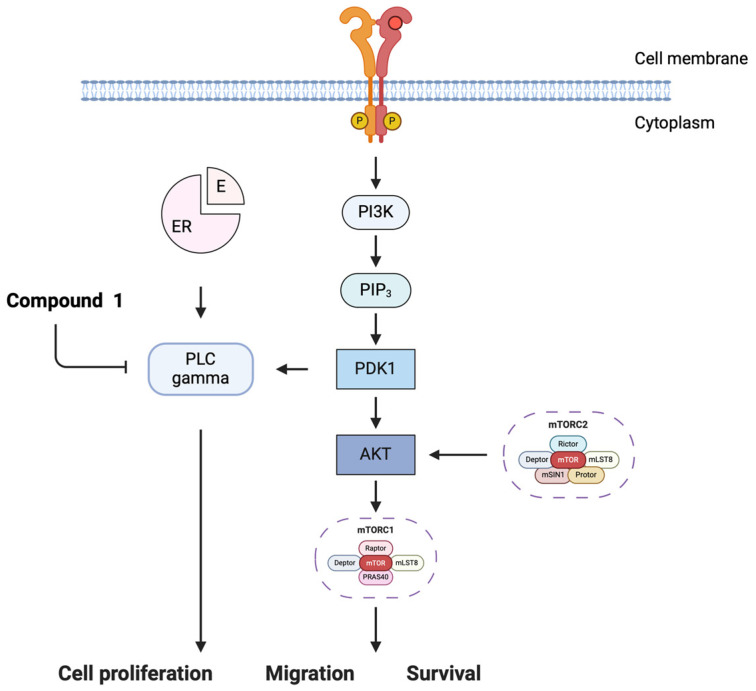
The interplay of growth factor and estrogen signaling in breast cancer cell proliferation, survival, and migration. Higher activation of PLCγ and mTOR is expected in MCF-7 cells containing ER, HER3, and a low level of HER2, which are absent in MDA-MB-231 cells. Compound **1**, as an inhibitor of PLCγ, is effective in lowering the percentage of MDA-MB-231 but not MCF-7 CSCs. Abbreviations: AKT, protein kinase B or Akt; Compound **1** or thieno[2,3-*b*]pyridine derivative, 3-amino-*N*-(3-chloro-2-methylphenyl)-5-oxo-5,6,7,8-tetrahydrothieno[2,3-*b*]quinoline-2-carboxamide; E, estrogen; ER, estrogen receptor; mTORC1 and mTORC2, mechanistic targets of rapamycin complex I and II; PDK1, 3-phosphoinositide-dependent kinase 1; PI3K, phosphoinositide 3-kinase; PIP_3_, phosphatidylinositol 3,4,5-trisphosphate; and PLC gamma, phospholipase C gamma. Created with BioRender.com.

Concerning our recent study [2], we can assume that thieno[2,3-*b*]pyridine-derivative-treated MCF-7 cells containing a low level of HER2 and a high level of HER3 are able to transduce the EGF signal using the HER2-HER3 heterodimer according to Balz et al., while MDA-MB-231 cells lack this ability [48,49]. Therefore, after treatment with thieno[2,3-b]pyridine derivative that inhibits PLC, MCF-7 cells maintain anaerobic metabolism and a fraction of cancer stem cells (CSCs), while MDA-MB-231 cells shift to aerobic metabolism and decrease the CSC fraction. In addition to EGF signaling, which results in PLCγ partial activation by binding phosphatidylinositol-3,4,5-trisphosphate (PIP_3_) via its N-terminal pH domain [50], these outcomes are expected due to progesterone and estrogen signaling, which are present in MCF-7 and absent in the MDA-MB-231 cell line (Figure 3). Signals from hormones bound to steroid receptors, such as estrogen receptors (ERs), can elicit the phosphorylation of PLCγ and activation of the anticipatory unfolded protein response. Yu et al. showed that Src kinase can trigger the activation of unfolded protein response during stress, enabling the tumor-protective mode in cancer cells [51].

Recently, Nguyen et al. proved that casein kinase 1 gamma 2 (CSNK1G2) was the most sensitive target to tamoxifen in MCF-7 cells, while tamoxifen was only slightly toxic in MDA-MB-231 cells [52]. ER silencing almost completely blocked tamoxifen sensitivity in MCF-7 cells. CSNK1G2 catalyzes the subsequent phosphorylation of phosphatidylinositol 3-kinase (PI3K)/AKT/mechanistic target of rapamycin (mTOR) signaling but not extracellular signal-regulated kinase (ERK) signaling in MCF-7 cells, while in MDA-MB-231 cells, CSNK1G2 activates ERK and slightly activates the PI3K/AKT/mTOR signaling pathway [52]. 

Different drugs are able to target breast cancer cells via mechanisms that involve lipid raft composition. Lipid-raft-associated PI3K/AKT signaling is a target of phenolic lipid gingerol from ginger in radio-resistant MDA-MB-231/IR cells, implicating a new raft-mediated treatment approach [53]. Membrane raft-localized EGFR expression is altered in MCF-7 and MDA-MB-231 cells by cholesterol metabolite 4-cholesten-3-one, which decreases breast cancer cell viability, reduces lipogenesis, and enhances liver X receptor-dependent cholesterol transporters [54]. Resveratrol treatment increases the fatty acid amount two- and four-fold in MCF-7 and MDA-MB-231 cells, which is accompanied by an increase in the expression of both flotillin-1 and flotillin-2 in breast tumors. Resveratrol induces a change in the pattern of flotillin distribution among lipid raft fractions in both cell lines and induces the nuclear translocation of flotillin-2 [55]. The incorporation of docosahexaenoic acid into whole-cell and lipid rafts differs between MCF-7 and MDA-MB-231 cell lines [56]. Gene expression profiles in MCF-7 and MDA-MB-231 differ concerning cholesterol homeostasis, implicating different lipid raft structures. In MCF-7 cells, miR-128 downregulation is favored because several genes involved in cholesterol synthesis and transport (*HMGCR*, *HMGCS1*, *SREBF1*, *SREBF2*, *CETP*, *LCAT*, and *LDLR*), MDR (*ABCC5* and *UGCG*), and cell signaling (*EGFR*, *IGF1R*, and *ESR1*) are downregulated, while the cholesterol efflux gene is increased (*ABCG1*) [57]. In the MDA-MB-231 cell line, it is demonstrated that adiponectin triggers fatty acid metabolic reprogramming that results in lipid depletion, triggering lipid raft disruption and apoptosis [58].

## 4. Aberrant Glycosphingolipid Metabolism as a Participant in Tumorigenesis and as a Possible Therapeutic Target

**Figure 4:**
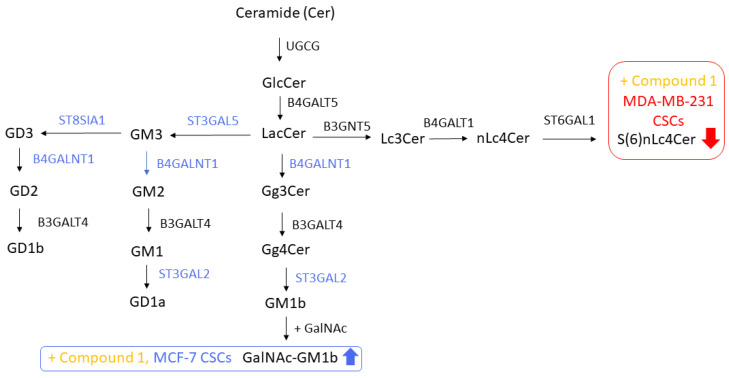
Synthesis of ganglioseries GSLs catalyzed by enzymes, written with blue letters, induced in breast CSCs [72,75] and of neolactoseries GSLs. Percentages of CSCs positive for red and blue framed GSLs were decreased and increased, respectively, after thieno[2,3-*b*]pyridine derivative (Compound **1**, yellow letters) treatment in MDA-MB-231 (red arrow) and MCF-7 (blue arrow) cells, respectively [2]. Abbreviations: In the ganglioside nomenclature, G = ganglioside, with the corresponding number of the sialic acid residues described with letters (M = mono, D = di), and the numbers denote the number of neutral sugar residues that are required to reach the number 5 (1 = GalGalNAcGalGlc, 2 = GalNAcGalGlc, 3 = GalGlc). Glycosidic residues: Gal = galactose, Glc = glucose, GlcNAc = N-acetylglucosamine, GalNAc = N-acetylgalactosamine. Neutral GSLs: Gg3Cer = gangliotriaosylceramide, Gg4Cer = gangliotetraosylceramide, Lc3Cer = lactotriaosylceramide, nLc4Cer = neolactotetraosylceramide. Acidic GSL: S(6)nLc4Cer = sialyl residue bound by α2-3 glycosidic bond to nLc4Cer. UGCG = UDP-glucose ceramide glycosyltransferase. In the nomenclature of other glycosyltransferases, the letter B = β-glycosidic bond, GALNT = N-acetylgalactosaminyltransferase, ST = sialyltransferase. Numbers within the name of β4-N-acetylgalactosaminyltransferase 1, B4GALNT1, represent the glycosidic bond between carbon C1 of the N-acetylgalactosaminyl residue and C4 of the galactosyl residue of LacCer in Gg3Cer, shown precisely in Figure 2.

GSLs influence cell proliferation, differentiation, migration, and survival due to their involvement in plasma membrane processes such as cell adhesion, membrane receptor activation, signaling, and endocytosis [59]. The silencing of the rate-limiting enzyme in GSL biosynthesis, glucosylceramide (GlcCer) synthase, encoded by the gene *UGCG* in humans, may thus interfere with cancer cell growth as shown in in vitro cell culture experiments (Figure 4) [60,61]. GlcCer synthase catalyzes the first step of GSL synthesis. Subsequently, GlcCer is elongated by Gal addition via enzyme B4GALT5, forming lactosylceramide (LacCer). The activation of B4GALT5 is a “pivotal” point of convergence for several signaling pathways initiated by growth factors, pro-inflammatory molecules, oxidative stress, diet, and cigarette smoking [62]. Further, formed LacCer can be the substrate of five enzymes: ST3GAL5, catalyzing the synthesis of the simplest negatively charged ganglioside GM3 (containing one sialic acid residue) (Figure 4); B4GALNT1, catalyzing formation of Gg3Cer, which is the first step of neutral ganglioseries GSL synthesis (Gg3, GalNAcβ4Galβ4Glcβ1Cer, shown in Figure 2; Gg4Cer, 4Galβ3GalNAcβ4Galβ4Glcβ1Cer) (Figure 4); A3GALT2 and A4GALT, catalyzing isogloboside and globoside synthesis, respectively; and B3GNT5, catalyzing (neo-)lactoseries GSL synthesis (Lc3Cer, GlcNAcβ3Galβ4Glcβ1Cer; nLc4Cer, Galβ4GlcNAcβ3Galβ4Glcβ1Cer; and S(6)nLc4Cer, IV^6^Neu5Ac Galβ4GlcNAcβ3Galβ4Glcβ1Cer (S, sialic acid residue or Neu5Ac) (Figure 4) [63,64]. 

Epithelial–mesenchymal transition (EMT) is a process in which epithelial cells lose their cell–cell contacts and apical–basal polarity, being thereby transformed to the mesenchymal phenotype with enhanced cell motility [65]. The EMT is often incomplete, and cells in the intermediate states display mixed epithelial and mesenchymal characteristics, giving them epithelial–mesenchymal plasticity properties [66]. EMT plays an important role in the initiation and development of cancer cell metastasis [67]. Gangliosides derived from the catalytic activity of ST3GAL5 (GM3, GM2, GD3, and GD2 from Figure 4) inhibit transforming growth factor-beta (TGFβ)-induced EMT via degradation of TGFβ type I receptor in normal mouse mammary gland epithelial NMuMG cells [64]. Consistent with this result, the addition of GM3 inhibits the migration of melanoma B16-F10 cells [68], while the finding of no influence of the treatment with exogenous GD3 on EMT is not consistent with the role of GD3 and GD2 in EMT maintenance [69]. Obviously, the effects of specific gangliosides on EMT vary between different cancer types. The inhibition of GD3 synthase (ST8SIA1, Figure 4) using shRNA or triptolide suppresses the invasion and motility of breast cancer cells and metastasis in mice, while its inhibition does not affect cell proliferation rates in vitro [69]. This finding is in accordance with the finding of inhibited c-Met signaling as a consequence of GD3 synthase inhibition and the known role of c-Met in the sustained proliferation and self-renewal properties of CSCs seeded to different tissues [69,70]. Globosides and ganglioseries inversely correlate and promote the transition between epithelial and mesenchymal cells of ovarian cancer [71]. CRISPR-Cas9-knocking-mediated truncation of endogenous E-cadherin in ovarian cancer cells induces EMT, which results in a decrease in globoside GSLs, while the deletion of ST8SIA1, which synthesizes GD3, induces mesenchymal–epithelial transition [71]. Levels of mRNA for enzymes encoded with ST3GAL2, ST3GAL5, ST8SIA1, and B4GALNT genes are elevated in breast CSCs, resulting in elevated GD2, GD3, GM2, and GD1a gangliosides accompanied by drastically reduced neutral GSLs Fuc-(n)Lc4Cer and the globoside Gb3 [72]. This finding of elevated ganglioside-synthesizing enzyme ST8SIA1 in breast CSCs is consistent with its elevation in ovarian cancer mesenchymal-like samples, predicting poor outcomes [71]. ST8SIA1 has also been suggested to be important for the initiation and maintenance of EMT in MDA-MB-231 breast cancer cells [73]. Namely, GD3 synthase (ST8SIA1) is the rate-limiting enzyme that converts the precursor GM3 to GD3 and further GD3 to GD2 and is known as a specific marker of triple-negative breast CSCs targeted with dinutuximab (Figure 4) [74]. Recently, Liang et al. confirmed in their model of EMT induction that GD3 synthase, together with its common upstream glycosyltransferase (GD2/GM2 synthase or B4GALNT1, Figure 4), maintains the stem cell phenotype in breast CSCs [75]. B4GALNT1 also induces motility and anchorage independence growth in melanoma [76]. 

Treatment with thieno[2,3-*b*]pyridine derivative, specifically 3-amino-*N*-(3-chloro-2-methylphenyl)-5-oxo-5,6,7,8-tetrahydrothieno[2,3-*b*]quinoline-2-carboxamide (Compound **1**), increases the percentage of ganglioseries GSL GalNAcGM1b (IV^3^Neu5Ac-Gg5; Gg5, GalNAcβ4Galβ3GalNAcβ4Galβ4Glcβ1Cer)-positive CSCs in the MCF-7 cell line and decreases the percentage of neolactoseries GSL S(6)nLc4Cer (IV^6^Neu5AcGalβ4GlcNAcβ3Galβ4Glcβ1Cer)-positive CSCs in the MDA-MB-231 breast cancer cell line [2]. 

The same experiment revealed a decreased percentage of Gg3Cer^+^ in MCF-7 CSCs, indicating that the addition of neuraminic acid (Neu5Ac) and two neutral sugar residues (Gal and GalNAc) to Gg3Cer, forming GalNAcGM1b ganglioside, favors the survival of MCF-7 CSCs. The whole sample of the MDA-MB-231 population (containing both CSCs and non-CSCs) shows a shift from anaerobic to aerobic metabolism. Studies of neolactoseries GSL expression in breast cancer cells are very rare. The deletion of β-1,3-N-acetyl-glucosaminyl-transferase 5 (B3GNT5), which synthesizes Lc3Cer, causes the loss of lacto/neo-lacto series GSLs and reduces α2-6 sialylation on N-glycoproteins in ovarian cancer cells [77]. The profiling of all known α2-6 sialyltransferase-encoding genes shows that the loss of α2-6 sialylation is due to the silencing of *ST6GAL1* expression in Δ*B3GNT5* cells, demonstrating that neolactoseries GSLs affect the complex network of *N*-glycosylation in ovarian cancer cells [77]. Breast CSCs show two-fold higher gene expression of B4GALT1, which catalyzes the synthesis of nLc4Cer, in comparison to non-CSCs [72]. In our earlier report, we showed five times higher expression of S(6)nLc4Cer per one non-treated non-CSC in comparison to CSCs of the MDA-MB-231 cell line [78], with a similar ratio being confirmed recently [2]. In spite of higher *B4GALT1* gene expression in CSCs reported by others [72], the percentage of S(6)nLc4Cer^+^CSCs decrease in the previous study is shown partially in Figure 4 [2]. This is most probably due to the lack of specific sugar residue substrates (S, sialic acid sugar residue, and Fuc, fucose) caused by thieno[2,3-*b*]pyridine derivative treatment.

MDA-MB-231 cells, mostly CD44^+^CD24^−^, represent triple-negative breast cancer (TNBC) cells, which lack three-hormone receptor expression; hence, there is an urgent need for their targetable markers [79]. CD44 is not a targetable marker owing to its expression in normal tissues. Ly et al. proposed ganglioside GD2 as a tumor-specific marker in TNBC patients due to its significantly higher expression in TNBC tissue compared to normal patient tissues [74]. The overwhelming majority of CD44^hi^CD24^lo^ cells express GD2 on the cell membrane [73]. CD44 mediates the aggregation of circulating MDA-MB-231 cells after their detachment from cancer tissue and the maintenance of their lipid raft integrity, which further enhances the stemness of aggregated cells [80].

Subsequent studies show the association of GD3 with EGFR and activated EGFR signaling in breast CSCs and breast cancer cell lines. ST8SIA1 (GD3 synthase) knockdown enhances cytotoxicity of gefitinib (an EGFR kinase inhibitor) in resistant MDA-MB468 cells, both in vitro and in vivo, indicating that ST8SIA1 contributes to gefitinib resistance in EGFR-positive breast cancer cells and its potential as a therapeutic target in drug-resistant breast cancers [81]. 

The enzymes NEU3, neuraminidase, and sialidase, which cleave glycoprotein and ganglioside neuraminic acid residue, enhance EGFR activation without affecting EGFR expression and act on its sialylation levels [82]. The indirect activation of EGFR is achieved via cleavage of neuraminic acid residue from the EGFR inhibitor, ganglioside GM3 [83]. Aberrant NEU3 expression is closely related to tumorigenesis [84]. NEU1 regulates proliferation, apoptosis, and expression in mammary carcinoma cells, but to date, only cadherins have been examined as their substrates and not gangliosides [85]. 

## 5. The Interplay of Glycosphingolipid Metabolism with Other Metabolic Pathways during Tumorigenesis and Therapeutical Attempts

**Figure 5:**
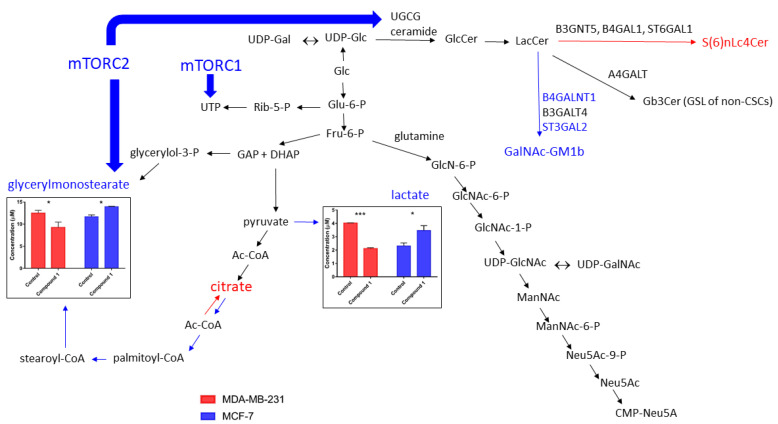
The interplay of glycosphingolipid metabolism with other metabolic pathways, which results in distinct metabolite and GSL expression findings in MDA-MB-231 and MCF-7 breast cancer cells after thieno[2,3-*b*]pyridine derivative treatment. Blue arrows and blue letters indicate the direction of metabolic reactions in MCF-7 cells, while red arrows and red letters indicate reactions in MDA-MB-231 cells. The red arrow from Ac-CoA to the citrate molecule indicates its catabolism in the citric acid cycle for aerobic energy production in the MDA-MB-231 cell line. Black arrows indicate common reactions for both cell lines; * *p* < 0.05; *** *p* < 0.001.

To explain the metabolic shift in MDA-MB-231 breast cancer cells, which we noticed after thieno[2,3-*b*]pyridine derivative treatment, it is necessary to review the interplay between GSL metabolism and other lipid and glucose metabolisms. Aerobic metabolism after derivative treatment, proved with lower lactate (Figure 5) and higher pyruvate levels [2], is accompanied by lower glycerylmonostearate (Figure 5), glycerylmonopalmitate, and myo-inositol levels [2]. In MCF-7 cells, derivative treatment increases lactate (Figure 5). Considering the pathway of glycosidic residue synthesis required for GSL synthesis, which shares fructose-6-P with glycolysis in parallel to different glycolysis patterns, a different response in GSL synthesis is expected. MCF-7 cells are able to obtain more glycosidic residues for GalNAcGM1b than MDA-MB-231 cells for S(6)nLc4Cer synthesis in the presence of thieno[2,3-*b*]pyridine derivative during cell cultivation [2]. Citrate transport from the mitochondria to the cytosol is elevated in conditions of elevated NADH in the case of anaerobic glycolysis [86]. Citrate is cleaved in cytosol, yielding acetyl-CoA, the precursor of palmitate and stearate, and consequently glycerylstearate, being increased in MCF-7 cells treated with thieno[2,3-*b*]pyridine derivative (Figure 5). Cancer cells take elevated levels of glucose in spite of decreased oxidative phosphorylation, which can yield maximal amounts of ATP. Their rate of glycolysis is increased due to their ability to produce ATP more quickly, optimizing energy production by maintaining a mix of metabolic capacities to support highly proliferated cancer cells [87]. Increased glucose uptake and lactate production via aerobic glycolysis is known as the Warburg effect [88]. Metabolic enzymes (hexokinase 2, pyruvate kinase type M2, and lactate dehydrogenase) and glucose and lactate (monocarboxylate) transporters are altered by the Warburg effect [89,90,91,92,93], which further increases the quantity of GM3 and sulphated SM4 GSLs required to maintain oncogenic GTPase KRAS in the plasma membrane enabling its signaling [94]. Among breast cancers, KRAS mRNA and GM3 expression have a prognostic role only in the luminal A subtype, which corresponds to the MCF-7 cell line [95,96,97]. GM3 has been identified and quantified in breast cancer serum with liquid chromatography–Fourier transform mass spectrometry (LC-FTMS) and liquid chromatography–mass spectrometry/mass spectrometry–multiple reaction monitoring (LC-MS/MS-MRM) [97]. More UTP and CTP, energy-rich molecules essential for glycosydic residue activation, and glycosydic residues (Figure 5) are produced in thieno[2,3-*b*]pyridine-derivative-treated MCF-7 cells, which could contribute to the synthesis of GalNAcGM1b^+^CSCs. On the other hand, aerobic glycolysis in treated MDA-MB-231 resulted in the statistically significant depletion of total CSCs, particularly S(6)nLc4Cer^+^CSCs [2]. 

Glucose availability and glycolytic metabolism are recognized as factors dictating glycosphingolipid levels in certain leukemia cells that take up elevated levels of glucose, resulting in increased glycolysis, activation of the pentose phosphate pathway, and biosynthesis of nucleotides, like UTP and CTP, required for GSL synthesis [98]. Increased flux into these pathways yields enough substrates for GSL formation, namely, fatty acyl-CoA for ceramide GSL portion, UDP-glucose, UDP-galactose, UDP-GlcNAc, UDP-GalNAc, and CMP-Neu5Ac (Figure 5). UDP-glucose ceramide glucosyltransferase (UGCG) is the key enzyme in GSL synthesis, and its induction generates GlcCer (Figure 5). It leads to increased proliferation in several cancer types, including proliferation of MCF-7 cells mediated with increased alanine–serine–cysteine transporter 2 (ASCT2) concentrations in GSL-enriched microdomains [99]. Although the acronym suggests otherwise, ASCT2 is a primary glutamine transporter in small-cell lung cancer cells located within lipid rafts rich in GD2 ganglioside [100]. Glutamine is necessary for the formation of aminoglycoside residues, such as GlcNAc and GalNAc, which are incorporated in GSLs (Figure 5), and its uptake is augmented by the induction of UGCG. This plays an important role in cell energy metabolism, particularly during the adaptation of breast cancer cells to poor nutritional supply. Glutamine serves to reinforce the following: 1, antioxidative capacity via conversion to a reducing agent (glutathione); 2, energy production through conversion to glutamate and further 2-oxo-glutarate, a metabolite of citric cycle; and 3, anti-apoptotic events by inducing proline levels [101]. The role of glutamine in the upregulation of glutathione biosynthesis, which maintains the homeostasis of reactive oxygen species, is also identified by Lampa et al. [102]. Glutamine is recognized as the potent activator of the mammalian target of rapamycin (mTOR), a conserved serine–threonine kinase. It is a part of the mTOR complex 1 (mTORC1), which helps to coordinate cell growth with nutritional status, and it is dysregulated in cancer cells [103]. In addition, glutamine contributes to the CSC GD2 ganglioside-positive phenotype in MDA-MB-231 cells, revealing that targeting of the glutamine transporters could be used as a complement to conventional chemotherapy in TNBC, as shown by the in vivo inhibition of tumor growth in a TNBC patient-derived xenograft mouse model [104]. The pathway of mTOR is highly activated in GD2+ breast CSCs [105]. Anti-GD2 antibody dinutuximab significantly decreases the adhesion and migration of MDA-MB-231 and SUM159 TNBC cells and inhibits mTOR signaling [74]. Dinutuximab significantly inhibits tumor growth in a patient-derived xenograft model in combination with activated natural killer cells [74]. Recently, hexahydrobenzo [4,5]thieno[2,3-*d*]pyrimidine derivatives have been described as potential anticancer agents that downregulate the PI3K/AKT/mTOR signaling pathway [106]. The thieno[2,3-*d*]pyrimidine-4(3H)-one derivative of benzimidazole [107], as well as thieno[2,3-*b*]pyridine derivative in our recent experiment [2], possess higher cytotoxicity against MDA-MB-231 than against MCF-7 cells. In addition to PLCδ1 inhibition, thieno[2,3-*b*]pyridines moderate multiple biological targets: copper-trafficking antioxidant 1 (ATOX1) protein, which induces the proliferation of cancer cells [108]; tyrosyl DNA phosphodiesterase 1 (TDP 1), which is a phospholipase D enzyme involved in repairing DNA damage [109]; the colchicine binding site of tubulin [110], which is a known anticancer target; and adenosine A2A receptor [111]. Due to the expectation of an overlap between the aforementioned targets, it is currently not known which receptor/enzyme is responsible for the noticed anticancer potential of the thieno[2,3-*b*]pyridines or whether a synergistic effect is responsible for the efficacy [112]. The in vivo administration of thieno[2,3-*b*]pyridine 3-amino-*N*-(3-chloro-2-methylphenyl)-5-oxo-5,6,7,8-tetrahydrothieno[2,3-*b*]quinoline-2-carboxamid reviewed in this article [2] had been earlier reported by Reynisson et al. A dose of 10 mg/Kg was injected intraperitoneally and was well tolerated by mice, with no acute reaction observed. The maximal plasma concentration achieved 30 min post-injection was 0.087 μmol/L, and the half-life was over 4 h [6]. The related compound thieno[2,3-d]pyrimidin is toxic against breast cancer MCF-7 cells (IC50 0.074 μM), with lower cytotoxicity towards mouse fibroblast 3T3 cells with an IC50 of 0.20 μM [107]. Doses of 15 different thieno[2,3-*b*]pyridines ranging from 0.75 to 133 mg/kg were proven to be active in the P388 leukemia tumor model (intraperitoneal) in B6D2F1 (BDF1) mice (https://pubchem.ncbi.nlm.nih.gov/bioassay/328#section=Data-Table, accessed on 21 August 2024).

The interaction of transglutaminase 2 and phospholipase C δ1 (PLCδ1) promotes Akt/mTORC1 activation and, consequently, autophagosome degradation in metastatic triple-negative breast cancer [113]. The combined signature of high transglutaminase 2/PLCδ1 levels correlates with lymph node metastasis of ER-negative breast cancer in patients with breast cancer. Blocking the protein–protein interaction of transglutaminase 2 and PLCδ1 inhibits lung metastatic colonies of TNBC cells in vivo [114]. Transglutaminase 2, known to mediate post-translational modifications of intracellular enzymes, is involved in the pathogenesis and progression of cancer. Its inhibition promotes apoptosis in MCF-7 and MDA-MB-231 cell lines, being particularly effective in decreasing MD-MB-231 cell migration and invasiveness [115]. 

The activation of mTORC1 results in nucleotide synthesis and proliferation, while mTORC2 promotes cancer-forming lipids essential for growth and energy production such as triacylglycerol (from glycerylmonostearate, Figure 5) and particularly sphingolipids (glucosylceramide, GlcCer; Figure 5) and glycerophospholipid (cardiolipin). In the same mTOR-driven mouse model, the prevention of hepatosteatosis from progressing to hepatocellular carcinoma is demonstrated by the inhibition of fatty acid or sphingolipid synthesis [116]. 

Knockdown of synaptopodin 2, a tumor suppressor inhibiting the transcription cofactors YAP/TAZ, enhances the PI3K/AKT/mTOR signaling pathway in MCF-7 and MDA-MB-231 cells, resulting in their migration and invasion [117]. Knockdown of caveolin-1, a principal structural component of caveolar membrane domains derived from lipid rafts, suppresses cancer development via the inhibition of PI3K/Akt/mTOR signaling, motility, and metastasis of breast carcinoma MDA-MB-231 cells [118]. The inactivation of mTOR and its dissociation from the lysosomal surface happens during starvation, causing transcription factor EB dephosphorylation, translocation to the nucleus, and the subsequent regulation of transcription of the genes involved in autophagy and lysosomal biogenesis [103,119,120]. Recently, Hartwig et al. noticed mTOR inactivation due to treatment with UDP-glucose ceramide glucosyltransferase inhibitors such as 1-phenyl-2-decanoylamino-3-morpholino-1-propanol, which causes the defective export of sphingolipids, lysobisphosphatidic acid, and cholesterol from lysosomes [121].

Signaling via the mTOR pathway in cancer is modulated by the Golgi-localized oncoprotein GOLPH3 [122], which also performs sorting of specific glycosyl transferases of GSL synthesis into vesicles for intra-Golgi retro-transport, enabling the cisternal maturation mechanism and controlling the sub-Golgi enzyme localization and its lysosomal degradation rate [123]. The decrease in GOLPH3 levels is accompanied by a decrease in GD1a and a concomitant increase in GM1 ganglioside expression at the surface of MCF-7 cells [124]. N-terminal domains of ST3GAL2 and B3GALT4 enzymes, which catalyze the last steps of GD1a and GM1 synthesis (Figure 3), respectively, interact to form the glycosyltransferase complex, which improves the enzymatic activity of one of the partners in the case of B3GALT4 [124,125]. GOLPH3 increases the glycosylation of cancer-relevant glycoproteins and regulates EGFR in glioblastoma cells via the modulation of its glycosylation [126] and has a role in MDA-MB-231 cells, sustaining their bioenergetic function and their mitochondrial fission [127]. 

Nohara et al. analyzed GSL expression in MDA-MB-231 and MCF-7 cells after their extraction from cells with organic solvents and, using thin-layer chromatography, reported four-fold higher ganglioside content in MDA-MB-231 cells compared to MCF-7 cells [128], which correlate with the values of geometric mean fluorescence intensities found by us using the flow cytometry method [2]. There is a lack of data in the literature concerning different GSL expressions in cell lines for each breast cancer subtype except for GD2 ganglioside. There is growing interest in GD2 investigation due to new discoveries emphasizing its roles in tumorigenesis and therapy [73,74]. Therefore, among all GSLs, only GD2 expression is described among different breast cancer subtypes. A low percent of the basal A subtype of basal-like 1 and basal-like 2 TNBC-subtype (MDA-MB-468, HCC1599 and HCC1806) and luminal (ER+ ZR-75-1, ER+PR+ MCF-7, Her2+ SKBR3, and ER+PR+Her2+ BT-474) cell lines are GD2-positive (up to 6%). The basal B subtype of mesenchymal, mesenchymal stem-like, and basal-like 1 TNBC-subtype TNBC cell lines (BT549, MDA-MB-231, SUM159, HCC38, and Hs 578T) are positive in more than 6% of the population, in 10% of MDA-MB-231, and in up to 99% in the HS 578T cell line [73,74,129]. One-third of several histologic types of breast carcinoma patients (386 in total) were positive for GD2. Positivity was significantly associated with the histologic subtype of the tumor, varying between metaplastic carcinomas (16%), triple-negative, invasive ductal carcinoma of no special type, ER-positive/HER2-negative, invasive lobular carcinoma, and triple-positive tumors (73%) [130]. Therefore, at least a proportion of these tumors may be therapeutically targeted with already available agents such as anti-GD2 antibodies and GD2-GD3 vaccines [131,132].

Serum GM3 is detected as a diagnostic marker of the luminal B subtype in comparison with other subtypes, and it is positively correlated with the Ki-67 status of patients, which indicates the number of mitoses [97]. On the other hand, GM3 expression is important in endothelial cells for the regulation of tumor angiogenesis by suppressing their invasion and oxidative stress tolerance. The GM3 knockout breast cancer mouse model shows an increase in tumor growth and angiogenesis [133]. The metabolomic profiling of blood-derived microvesicles in breast cancer patients reveals that sphingomyelin SM (OH) C16:1 is among eight significant metabolites differentiating breast cancer patients from controls [134]. The combination of the six sphingomyelins with 18 phosphatidylcholines differentiates between luminal A and B breast cancer subtypes [134]. GSLs within blood-derived microvesicles were not analyzed. Nevertheless, due to the distinct sphingomyelin profile, we can also assume that some of the GSLs can be used to differentiate breast cancer subtypes. Sphingolipid and ether lipid expression patterns are altered in breast cancer cells in an estrogen-receptor-dependent manner, being strongly upregulated in SKBr3 (ER-negative and G-protein coupled estrogen receptor 1, GPER1, positive) in comparison to T47D (ER + and GPER1-) and MCF-7 (ER + and GPER1+) cells [135]. It is important to focus on more than one lipid class in breast cancer cells because co-expression of different lipids could influence tumorigenesis and the achievement of improved breast cancer treatment outcomes [135].

## 6. Conclusions and Perspectives

Glycosphingolipid expression is strongly entwined with cell metabolism. GSLs cause specific shifts of metabolism during tumorigenesis via their effects on growth factor receptors and glutamine transporter (alanine–serine–cysteine transporter 2) concentrations in GSL-enriched plasma membrane microdomains. On the other hand, treatments of cancer stem cells can result in distinct GSL expression, which contributes to the decrease in CSCs. 

The finding of higher activity of B3GALT4 and higher GM1 ganglioside level caused by the decrease in GOLPH3 level deserves particular attention due to the expected potential increase in GD1b synthesized by the same enzyme and decrease in its substrate GD2 (Figure 3). Recent clinical evidence of the worse 5-year recurrence-free survival rates among patients with GD2-positive TNBC in comparison to patients with GD2-negative TNBC brings the novel strategies of GD2 synthesis inhibition into focus within TNBC research. The novel thieno[2,3-*b*]pyridine anticancer compound, being able to target TNBC cells that express glycosphingolipid S(6)nLc4Cer on their surface and diminish the Warburg effect that leads to lower total CSCs, deserves further investigation. Considering that two cell lines (MDA-MB-468 and HCC1599) of the basal A subtype of TNBC basal-like 1 subtype have a low GD2-positive fraction, thieno[2,3-*b*]pyridine needs to be proved in vivo as a therapy for patients who do not respond to anti-GD2 antibodies.

## Data Availability

No new data were created.
